# WPAN and IoT Enabled Automation to Authenticate Ignition of Vehicle in Perspective of Smart Cities

**DOI:** 10.3390/s21217031

**Published:** 2021-10-21

**Authors:** Anita Gehlot, Rajesh Singh, Piyush Kuchhal, Adesh Kumar, Aman Singh, Khalid Alsubhi, Muhammad Ibrahim, Santos Gracia Villar, Jose Brenosa

**Affiliations:** 1 Department of Electronics and Electrical Engineering, Lovely Professional University, Phagwara 144411, Punjab, India; anita.23401@lpu.co.in (A.G.); rajesh.23402@lpu.co.in (R.S.); 2 Department of Physics, University of Petroleum and Energy Studies, Dehradun 248007, Uttarakhand, India; pkuchhal@ddn.upes.ac.in; 3 Department of Electronics and Electrical Engineering, University of Petroleum and Energy Studies, Dehradun 248007, Uttarakhand, India; adesh.kumar@ddn.upes.ac.in; 4 School of Computer Science and Engineering, Lovely Professional University, Phagwara 144411, Punjab, India; 5 Faculty of Computing and Information Technology, King Abdulaziz University, Jeddah 37848, Saudi Arabia; kalsubhi@kau.edu.sa; 6 Department of Information Technology, University of Haripur, Haripur 22620, Pakistan; 7 Universidad Europea del Atlántico, Isabel Torres 21, 39011 Santander, Spain; santos.gracia@uneatlantico.es (S.G.V.); josemanuel.brenosa@uneatlantico.es (J.B.); 8 Project Department, Universidade Internacional do Cuanza Bairro Kaluanda, EN 250 Cuito, Bié, Angola; 9 Department of Project Management, Universidad Internacional Iberoamericana, Campeche C.P. 24560, Mexico

**Keywords:** ESP8266, helmet, two-wheeler, Flex sensor, internet of things, RFID, 2.4 GHz RF communication

## Abstract

Currently, two-wheelers are the most popular mode of transportation, driven by the majority the people. Research by the World Health Organization (WHO) identifies that most two-wheeler deaths are caused due to not wearing a helmet. However, the advancement in sensors and wireless communication technology empowers one to monitor physical things such as helmets through wireless technology. Motivated by these aspects, this article proposes a wireless personal network and an Internet of Things assisted system for automating the ignition of two-wheelers with authorization and authentication through the helmet. The authentication and authorization are realized with the assistance of a helmet node and a two-wheeler node based on 2.4 GHz RF communication. The helmet node is embedded with three flex sensors utilized to experiment with different age groups and under different temperature conditions. The statistical data collected during the experiment are utilized to identify the appropriate threshold value through a *t*-test hypothesis for igniting the two-wheelers. The threshold value obtained after the *t*-test is logged in the helmet node for initiating the communication with the two-wheeler node. The pairing of the helmet node along with the RFID key is achieved through 2.4 GHZ RF communication. During real-time implementation, the helmet node updates the status to the server and LABVIEW data logger, after wearing the helmet. Along with the customization of hardware, a LABVIEW data logger is designed to visualize the data on the server side.

## 1. Introduction

The two-wheeler is the significant transport mode that is adopted by the majority of the population of India due to the lack of alternative affordable transport facilities [1]. At present, 70% of India’s vehicles are two-wheelers, followed by cars at less than 13% of the total number of vehicles on the road. Furthermore, the production of two-wheelers was over an 18-million-unit figure in India during the fiscal year 2021 [2]. With this two-wheeler production, security and safety are the main concern as six two-wheeler riders die every hour in accidents, and 37% of deaths are accounted for by two-wheeler-related incidents [3]. According to the WHO, the main reason for deaths related to two-wheelers is not wearing helmets, or wearing faulty helmets [4]. Even the WHO’s research has concluded that wearing a helmet would reduce fatal and serious head injuries by 20% to 45%.

Generally, during two-wheeler transportation, structural protection is necessary for protecting the body from sustaining injury [5]. Especially for two-wheelers, the riders are vulnerable to head injuries, so it is suggested to wear a helmet for reducing injuries during accidents. Fundamentally, for every product, certain standards are formulated for delivering effective, safe, and reliable services to the users. Concerning the helmets, the Government of India (GoI) is implementing the Bureau of Indian Standards (BIS) [6]. Yet, these approaches have not reduced the number of accidents because the population are still not wearing helmets while riding. However, these challenges can be overcome by integrating advanced and emerging technology aimed at encouraging people to wear a helmet during riding. An embedded system is one of the prominent technological systems widely implemented in cars and two-wheelers in, for example, the anti-lock braking system, navigation system, adaptive cruise control, and ignition system [6]. Global positioning systems (GPS) and global systems for mobile communication (GSM) are integrated to detect the location of the vehicle through short message services (SMS) [7,8]. The implemented system in two-wheelers is operated on the battery power, so it is suggested that the implementation of GSM and GPS be avoided due to high energy consumption. Additionally, for receiving the alerts, the GSM-based SIM needs to be prepaid.

To overcome these constraints, implementation of a wireless personal network (WPAN) has gained attention for establishing the communication between the user and the vehicle [9]. Bluetooth and 2.4 GHz-based ZigBee are the prominent technologies that are integrated for establishing the WPAN [10,11]. Bluetooth and 2.4 GHz-based ZigBee also consume low power with low data rates and short-range transmission of data [12]. These communication protocols assist the two-wheeler user in detecting helmets, authenticating the vehicle, and automatic ignition with minimum resource. The interconnection of the two-wheeler with IoT initiates to implement the real-time tracking and further the real-time data generation from the sensors is utilized through artificial intelligence [13].

With this motivation, we are implementing a hybrid system that enhances the security of two-wheelers with automatic authentication and ignition with 2.4 GHz-based Zigbee RF communication. The helmet node in the helmet detects whether the helmet is being properly worn by the user. The helmet node with the assistance of a 2.4 GHz RF modem transmits the data to the two-wheeler node that is embedded within the vehicle. The two-wheeler activates the radio frequency identification (RFID) reader to authenticate the user for starting the ignition. Additionally, we have integrated the Wi-Fi module in the two-wheeler node for realizing the IoT enabled system [14]. The main contribution of the study is as follows:

- Implementation of an IoT enabled system for realizing the authentication of the user in order to ignite the two-wheeler with 2.4 GHz wireless communication and radio frequency identification (RFID) technology.
- An evaluation is performed for the exact placement of flex sensors inside the helmet node to match the appropriate pressure exerting points.
- Validating the authentication of the user for initiating the ignition is achieved with the assistance of an RFID reader and flex sensors in the helmet node.
- The threshold value of the two-wheeler vehicle is observed as ‘212’ or 1.03 V through *t*-test analysis.
- The current consumption obtained for the two-wheeler node is 188 mA and the server is 98 mA.

The organization of the study is as follows: Section 2 covers the literature review; Section 3 covers the complete description of the working mechanism of the system with the help of block diagrams of the two-wheeler section and helmet section; Section 4 covers the flow diagram of the helmet section, two-wheeler section, and LabVIEW; Section 5 describes the complete methodology to develop the system; Section 6 explains the analysis of the experimental results of the developed system using a *t*-test; Section 7 includes major outcomes with a conclusion and discussion of the future scope of the system.

## 2. Review of Literature

The system based on RFID and FSR is built so not to enable the rider to start a two-wheeler without wearing a helmet, with the vehicle ignition being controlled by a cumulative decision by the RFID reader in the car and FSR in the helmet [15,16]. A technique is proposed for developing a safety system that combines a smart helmet and an intelligent bike to minimize the chances of two-wheeler accidents, bike theft, and drunk driving cases [17]. A smart ignition system is implemented to detect the alcohol level of the two-wheelers rider based on GSM, Bluetooth, and Node MCU [18]. An intelligent traffic surveillance system is implemented for recognizing motorcyclists from video feeds in real-time using a convolutionary object detector [19,20].

A data acquisition system for measuring the pressure inside the helmet is created. [21]. A system with RFID and FSR sensors is implemented for avoiding accidents due to negligence of two-wheeler riders [22,23]. This study is relevant for two-wheelers like bicycles, motorcycles, scooters, and also for skateboards. Results show that the riders without helmets sustain more serious injuries [24]. This study proposes a system that creates a collaborated environment of helmet and fingerprint sensor which ignite the bike only after wearing the helmet by the rider [25]. An environmental pressure monitoring system is used to identify the tidal range in danger zones with the help of a piezoresistive pressure sensor and Zigbee-based Wireless Sensor Networks (WSNs) [26]. Wireless monitoring is used for industrial applications at remote locations with a combination of embedded systems and Zigbee [27]. A wide variation of the sensor data characteristics inside the helmet and the average value of the data from these sensors can be taken as the reference value [28].

A pressure sensor-based system ignites the engine after getting a value greater than the predefined values in the program, with known pressure values taken and used [29]. LabVIEW GUI was developed to display all pressure sensor values as real-time bar graphs or as analog pressure versus time curves. An invention related to the wireless link between portable entertainment systems and two-wheelers and helmets is proposed, and the helmet is integrated with four FSR sensors for the detection of helmet-wearing [30,31]. Safety equipment is equipped with FSR sensors to ensure the person wears a helmet. These sensors are implemented with the two parameters which are maximum resistance and minimum resistance [32]. The accelerometer measurements are communicated to the Processor, where it continually checks for irregular deviations and, if an accident happens, the relevant information is communicated to the emergency contacts through a cloud-based service [33].

A smart helmet with a single integrated fiber Bragg grating (FBG) sensor was designed for real-time detection of blunt-force impact incidents to helmets [34]. WPAN and LabVIEW-based systems were designed for monitoring the water quality at home through RF modem and the designed system is capable of giving the early warning for the contaminated water [35]. A smart alert technique is implemented for establishing an intelligent vehicle that enables automatic avoidance of accidents made by the drowsy driver using Raspberry pi [36]. IoT enabled Arduino and Node MCU-based vehicle tracking is implemented for alerting incidents of the accident to the prescribed users that preset during installation of the system and also authentication of the user is achieved using their fingerprint [37]. From the literature review, it is concluded that the system with multiple features like helmet authorization and authentication of the user is very limited. Moreover, the WPAN guides us to the implementation of a low power-enabled XBee protocol for the transmission of data within a short-range.

Table 1 presents the comparative analysis of the proposed study with previous studies based on vehicle security. The comparison is done based on parameters such as function, real-time hardware, ignition mechanism, user authentication, proof of concept, communication, and the threshold value for detection. In previous studies, the security of the vehicle is implemented with the assistance of different approaches, however, during real-time implementation, the main factor that needs to be considered is power consumption. The power consumption is managed through customizing the hardware with necessary components that are to be part of the system. In this study, the real-time hardware is designed with a low powered communication protocol i.e., 2.4 GHz RF. The ignition mechanism and user authentication of the vehicle are achieved with a flex sensor and RFID technology. Moreover, the placement of the sensors is also evaluated and finalized based on the helmet standards. Statistical analysis is carried out with a *t*-test for determining the threshold value that is useful for detecting the helmet. Furthermore, the proposed system is implemented in a real-time environment on a two-wheeler vehicle

## 3. System Description

**Figure 1:**
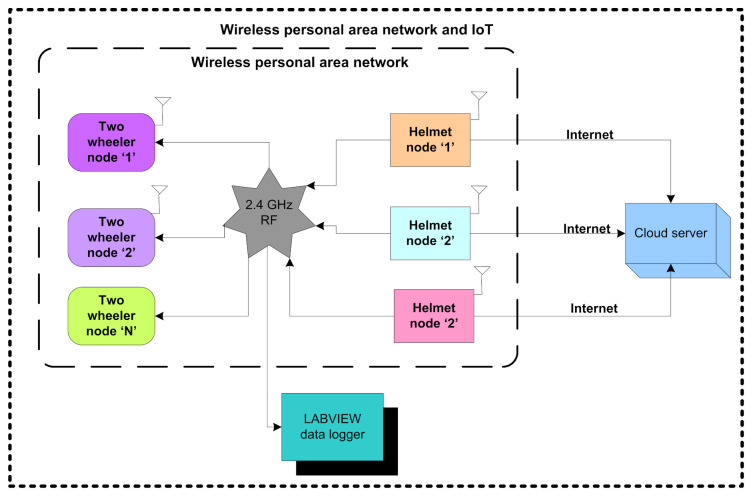
WPAN and IoT-based architecture.

In this section, the proposed architecture for authorized ignition based on 2.4 GHz communication and RFID technology is presented and shown in Figure 1. The proposed architecture is the integration of three individual components namely: the helmet node, two-wheeler node, and cloud server. The proposed architecture enables the implementation of an authentic approach with the helmet node. The three flex sensors placed in the helmet node authorize that an individual is wearing the helmet.

The helmet node also confirms to the two-wheeler node that the person is wearing the helmet through 2.4 GHz communication. The receiver unit of the two-wheeler node receives the confirmation through 2.4 GHz communication and activates an RFID reader embedded in the two-wheeler node to confirm the authentication of the person. After the completion of the authentication, the two-wheeler node ignites the two-wheeler and sends the information about the ignition to the helmet node. The node MCU embedded with the helmet node logs the sensory information, authentication, and ignition to the cloud server, and also sends to the LABVIEW data logger through 2.4 GHz communication.

**Figure 2:**
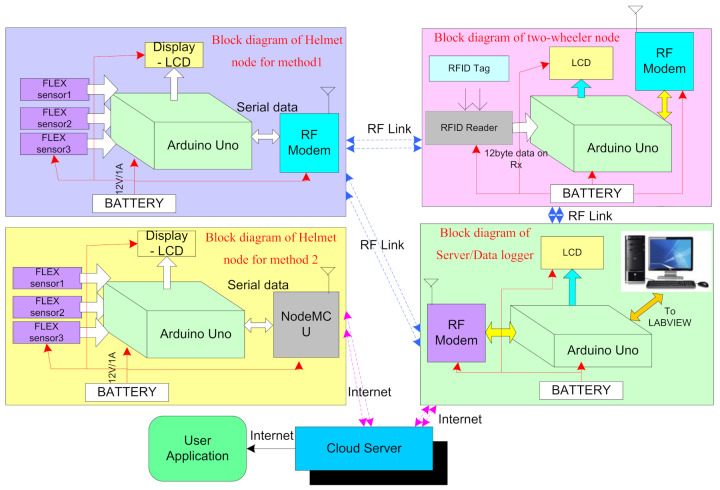
Working flow of the system.

The system is developed in three sections, namely, the helmet node, the two-wheeler node, and the server/data logger as shown in Figure 2. The helmet node (transmitter section) comprises of three flex sensors, a controller unit, a power supply (rechargeable battery), a serial port (to charge the battery), and an RF modem. The two-wheeler node (receiver section) comprises of an RF modem, controller unit, RFID reader (to authentic the user), battery, and relay (to ignite the vehicle). The server/datalogger comprises of an RF modem, controller unit, power supply (battery), and USB to a serial port (to interface with PC/LabVIEW).

The helmet node (HN) is transmitting the average value of flex sensors to the two-wheeler node (TWN) and the data logger through an RF modem for analysis purposes. After analysis, the data received from the helmet node is shared with the two-wheeler node and all threshold values are set accordingly. By following the same procedure, the respective authorities set the threshold values for helmet detection in the helmet node. In the initial stage of installing the system, depending upon the user, the threshold values are set in the helmet node through user application. The authentication details of the user application are also provided to the user for modifying the threshold values in future. The user application based on the cloud server also visualizes the real-time sensor values (Figure 2).

### 3.1. Helmet Node (Transmitter Section)

The helmet node is designed in two different methods as shown in the Figure 2. In method 1, the helmet node is embedded with a 2.4 GHz RF modem for transmitting data to the datalogger and two-wheeler node. The block diagram of method 1 and method 2 is shown in Figure 2. In method 2, the system is embedded with Node MCU to transmit data from the helmet node to a cloud server as shown in Figure 2. Node MCU based on IEEE 802.11 b/g/n provides internet to the helmet. The helmet node also comprises of three flex sensors that generate values corresponding to the change in angle and this value is transmitted to the controller unit as shown in the block diagram of helmet node for method 1. The data recorded in the cloud server can be beneficial for data analysis. Furthermore, it is useful for analyzing the data in order to detect the accident and generate an alert signal to the nearest hospital or police station for quick help using the coordinates of the user. In the current study, the data is analyzed only for finding the threshold value of sensors to ignite the vehicle. The future scope of this research includes calculating the threshold value of the sensors to detect an accident.

### 3.2. Two-Wheeler Node (Receiver Section)

The block diagram for the two-wheeler node is shown Figure 2. In the receiver section, the RF Modem receives the data transmitted by the helmet. This data contains the average value of all the sensors inside the helmet. When a bike rider wears a helmet, the helmet section sends the average value to the two-wheeler node. Then the RFID card is to be swiped and the controller unit performs an AND operation on these two signals compared with the predefined values. If both data match with threshold values, then a two-wheeler is ignited through the relay.

### 3.3. Server/Data Logger

Sensory data is analyzed with two methods. One is to analyze data for checking the working of helmet node with LabVIEW GUI and the other is to analyze sensory data on a cloud server. The data logger shown in Figure 2 comprises a controller unit, LCD, and RF modem. It is used to analyze the sensory data and system working with LabVIEW GUI. The sensory data is transmitted on the cloud server on Thingspeak.com, which is free to access. It can be accessed from anywhere in the world. This is designed for the method 2 helmet node. In the helmet node, the output value from the sensors is collected and the average value is transmitted to the two-wheeler node through the RF Modem.

### 3.4. Interference of RF Signal

**Figure 3:**
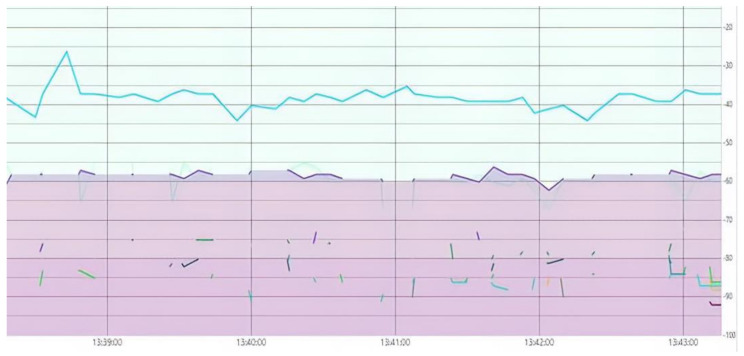
RSSI- based interference test.

In the industrial scientific and medical (ISM) band, the following wireless technologies namely Bluetooth, Wi-Fi, and Zigbee are operated on a 2.4 GHz frequency band. The interference of these wireless technologies is tested by using a radiofrequency Received Signal Strength Indicator (RSSI), and channel state information (CSI) techniques [38]. A received signal strength indicator (RSSI) is utilized for testing the interference of radio frequency signals of same frequency band i.e., 2.4 GHz [39]. In RSSI, the coarse-grained activity recognition is achieved as it falls in the time domain. The interference test is carried out by considering the RSSI of the various devices on the same frequency spectrum. Furthermore, the device indicted to have the lowest RSSI (closer to 0 dB), and thus the other interference in that spectrum is not subjected to overlap with our signal or message, as shown in Figure 3, where the blue signal represents the RSSI of our concerned device and the other signal interference is represented in the violet color.

**Figure 4:**
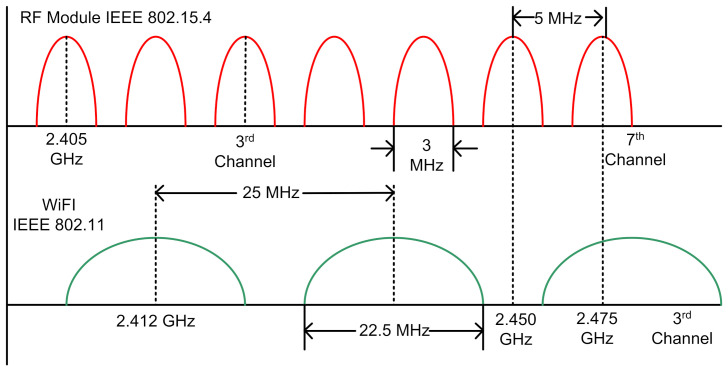
Radiofrequency plot of Zigbee and Wi-Fi.

Moreover, as shown in Figure 4, the plot shows the spectrums of corresponding Wi-Fi and 2.4 GHz RF. As both the Wi-Fi and 2.4 GHz-based Zigbee RF works through spread spectrum transceiver methodology where they never continue to operate on the same radio channel indefinitely. Furthermore, they tend to occupy the bands of frequencies being the center frequency band as the reference to the radio channel assignment. Wi-Fi occupies a considerably large band channel thereby occupying more RF spectrum per channel than RF. The channel space between these allows them to coexist without interfering.

In addition, in a scenario of close gaps between the 2.4 GHz based Zigbee RF and other present Wi-Fi sources, the system utilizes Packet Traffic Arbitration (PTA) methodology which works in the pathway of Request to send (RTS)/Clear to send (CTS) as follows:

- The transmitter node of the helmet first sends a request along with a priority.
- The receiver device at the vehicle node performs channel activity detection and sends back the response to the transmitter node about the channel status (i.e., empty, filled).
- On receipt of CTS from the receiver device depicting that the transmission channel is empty and no one is currently using it, the transmitter node sends its data.
- This is a kind of RTS/CTS hand-shaking methodology that is widely used in various systems to avoid the overlapping of data and interference.

## 4. Methodology

**Figure 5:**
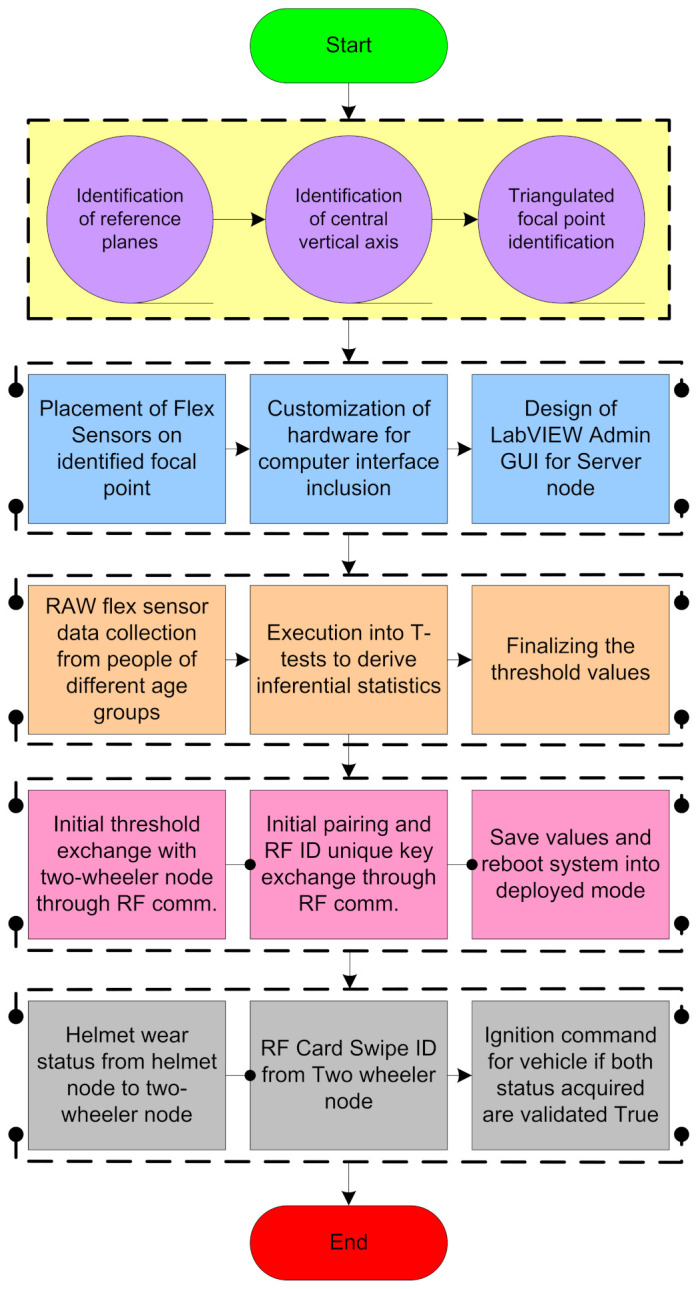
Methodology for the experimental test.

In this section, we present the methodology of the reading and testing of the flex sensor. Moreover, the flow diagram of the helmet node and two-wheeler node that are discussed in Section 4 explains the working of the proposed system. Figure 5 presents the methodology in detail, including the system implementation from the placement of the sensor to its real-time implementation.

In the initial stage of system implementation, the placement of the sensors is carried out in the helmet. In the placement of the flex sensors, the identification of reference planes and the central vertical axis is evaluated with a triangulated focal point inside the helmet for the appropriate placement of flex sensors at that point where the pressure exerted is greater on the head while wearing the helmet. The three different points are mainly considered because these are the points where the pressure is exerted after wearing the helmet. The three points are B (front side of the head), Z&X (right and left side of head slightly above to ears), and R (backside of head). The flex sensors are placed inside the helmet above these three points. After the placement of the sensors inside the helmet, in the next step, we developed customized hardware for enabling the authenticated ignition. Along with the customization of hardware, a LABVIEW datalogger is designed to visualize the data on the server side. The helmet that is embedded with three flex sensors has been experimented with accounting for different age groups under different temperature conditions.

The statistical data received during the experiment is utilized for deciding the appropriate threshold value for igniting the two-wheelers. The threshold value is confirmed by applying the *t*-test hypothesis. The threshold value obtained after the *t*-test is logged in the helmet node for initiating the communication with the two-wheeler node. The pairing of the helmet node along with the RFID key is achieved through 2.4 GHZ RF communication. During real-time implementation, the helmet node updates its status to the server and the LABVIEW datalogger after the helmet is worn. Ignition is command for the vehicle if both statuses acquired are validated to be true. In the following section, the explanation of the reading and placement of flex sensors along with the RFID code extraction is discussed.

### 4.1. Read the Flex Sensor

**Figure 6:**
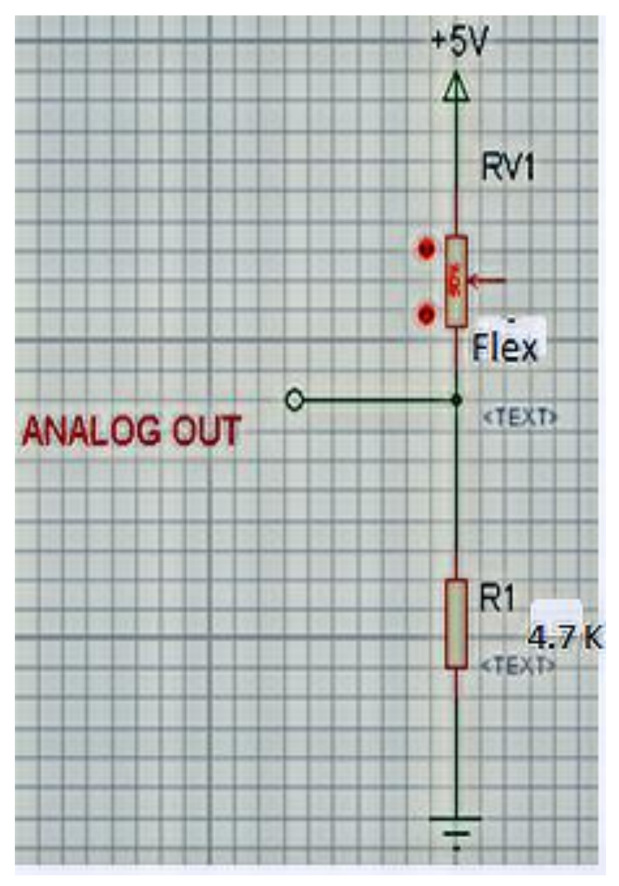
Arrangement for reading the flex sensor.

A flex sensor is used for sensing change strain exerted between the head and helmet when the driver wears it. Flex is a low-cost sensor, and it is simple to use. It is a variable resistor, the value of which varies according to change in strain. The value of resistance decreases with an increase in the change in angle of the sensor. Figure 6 shows the arrangement for reading the flex sensor.

Output voltage for flex is given as equation below-

(1) $$\mathrm{flex}\left(V\right)\;=\mathrm{flexADC}*\left(\frac{\mathrm{Vcc}}{1023}\right)$$

(2) $$\mathrm{flex}\left(V\right)\;=\;\left(\frac{\mathrm{flex}\left(R\right)}{R+\mathrm{flex}\left(R\right)}\right)*\mathrm{Vcc}$$

(3) $$\mathrm{flex}\left(R\right)\;=\;R\;*\;\left(\frac{\mathrm{Vcc}}{\mathrm{flex}\left(V\right)-1}\right)$$

where R = 4.7 K. R implies resistance connected to flex, Flex(R) implies resistance by Flex sensor, Vcc implies Input Voltage (V) and V implies Analog output.

#### Placement of the Flex Sensors

**Figure 7:**
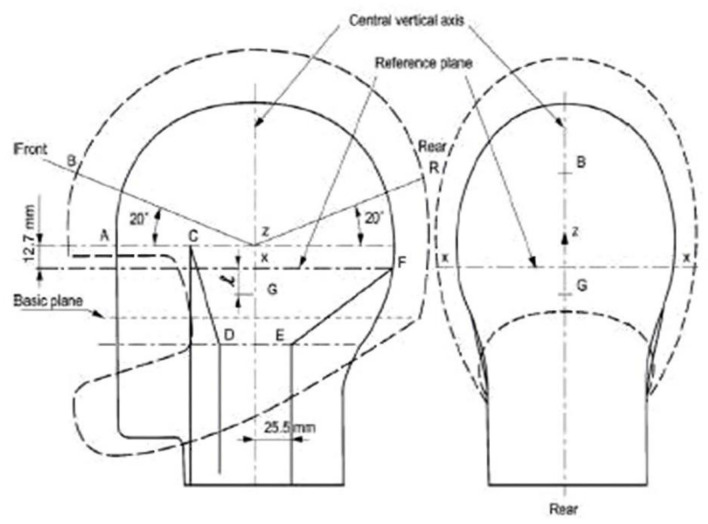
Placement of the flex sensors.

Helmet standard agencies identified four pressure points in the helmet where a maximum change in pressure is exerted when an accident happens. The points are B (front side of the head), Z&X (right and left side of head slightly above to ears), and R (backside of head) as shown in Figure 7. While wearing helmet, three points B, Z and X exert pressure, so these three points are considered for the placement of flex sensors so that it can be verified whether the driver is wearing a helmet or not. While designing a microcontroller chip, four analog sensor input pins are considered so that the same microcontroller can be used in the future to analyze accident impact on the head.

### 4.2. RFID Code Extraction

**Figure 8:**
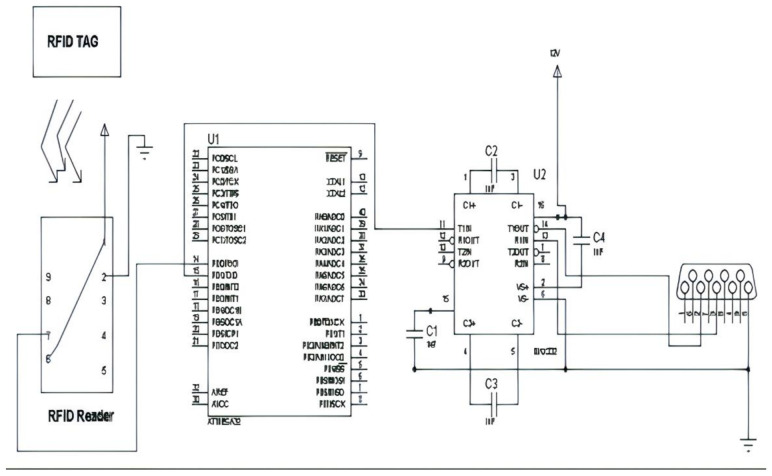
Circuit diagram for RFID code extraction.

To identify the authorized person, RFID tags are used with an RFID reader which is placed at a two-wheeler node. Each RFID tag has a unique twelve-byte code that needs to be extracted from the tag and the same needs to write in the program. Then, the controller has to identify a predefined code when the user swipes the RFID tag on the RFID reader. A small circuit needs to be developed for extracting the code from the RFID tag, as shown in Figure 8. The extracted code can be checked on software terminal V1.9.

Connect pins no. 2 and 3 of the DB9 connector to pins 14 (R2-IN) and 13 (T2-OUT) of MAX232 IC, respectively. Connect pin 11(T2-IN) of MAX232 to 15 (TX) of Atmega32. Connect a capacitor between 1 and 3 of MAX232 where (+) the terminal of the capacitor is connected to pin no. 1 and 3 with the (−) terminal of the capacitor. Connect a capacitor between 4 and 5 of MAX232 where the (+) terminal of the capacitor is connected to pin no. 4 and 5 with the (−) terminal of the capacitor. Connect a capacitor between 2 and 16 of MAX232 where the (+) terminal of the capacitor is connected to pin no. 2 and 16 with the (−) terminal of the capacitor. Connect a capacitor between 6 and 15 of MAX232 where the (+) terminal of the capacitor is connected to pin no. 6 and 15 with the (−) terminal of the capacitor. Connect pin no. 7 (data) of RFID to pin no. 14(RX) of Atmega32. Connect pins 1 and 6 of RFID to Vcc(+5 V) and pin no. 2 to Ground.

## 5. Flow Diagram

**Figure 9:**
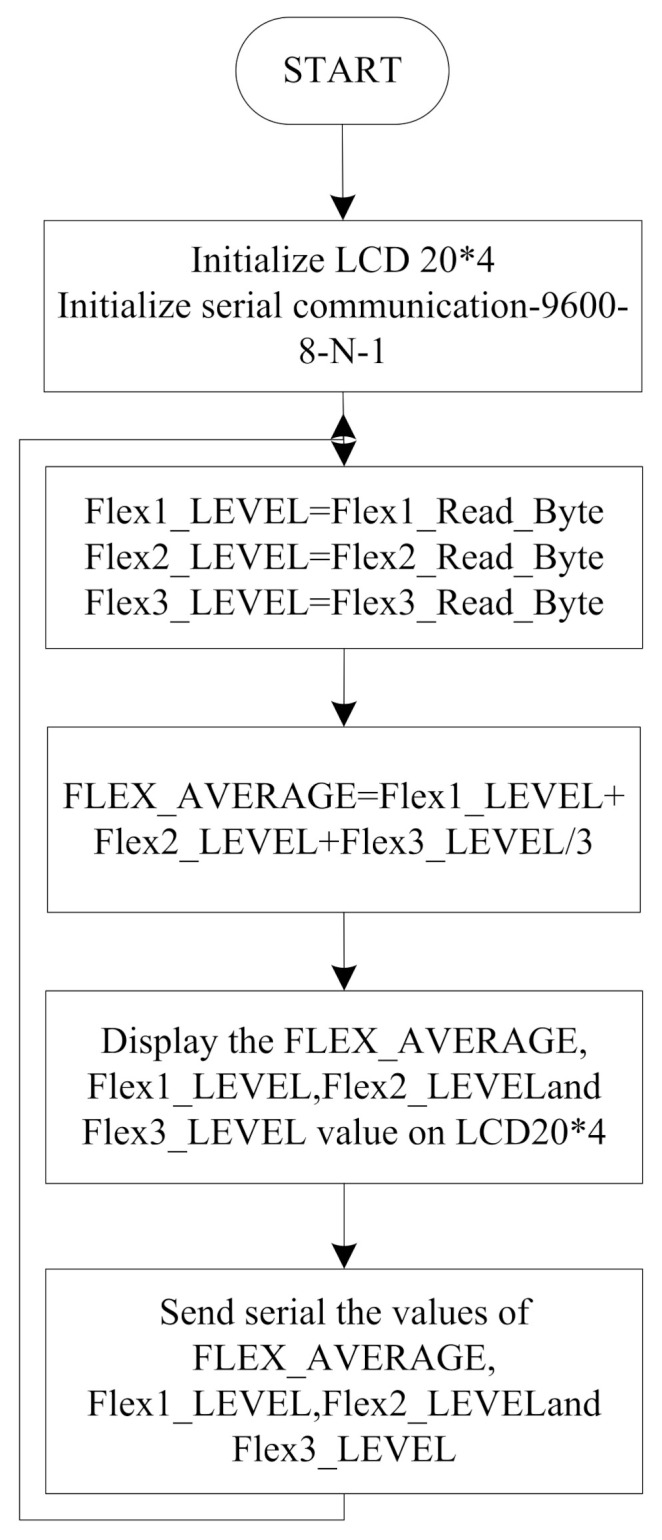
Flow chart for the helmet node.

In this section, we discuss the flow of the helmet node, two-wheeler node, and server as follows:

Figure 9 shows the flow diagram of the helmet node. The functions for LCD and serial communication are initialized. After the initialization, the system will wait for the signals from three flex sensors placed in the helmet. The average value of the three sensor outputs is taken, which is displayed on the LCD for experiment purposes and sent to the two-wheeler node through serial communication at a baud rate of 9600 bps. To send data on the cloud server node, MCU is connected to Arduino UNO and receives the data on www.thingspeak.com (accessed on 11 October 2021).

**Figure 10:**
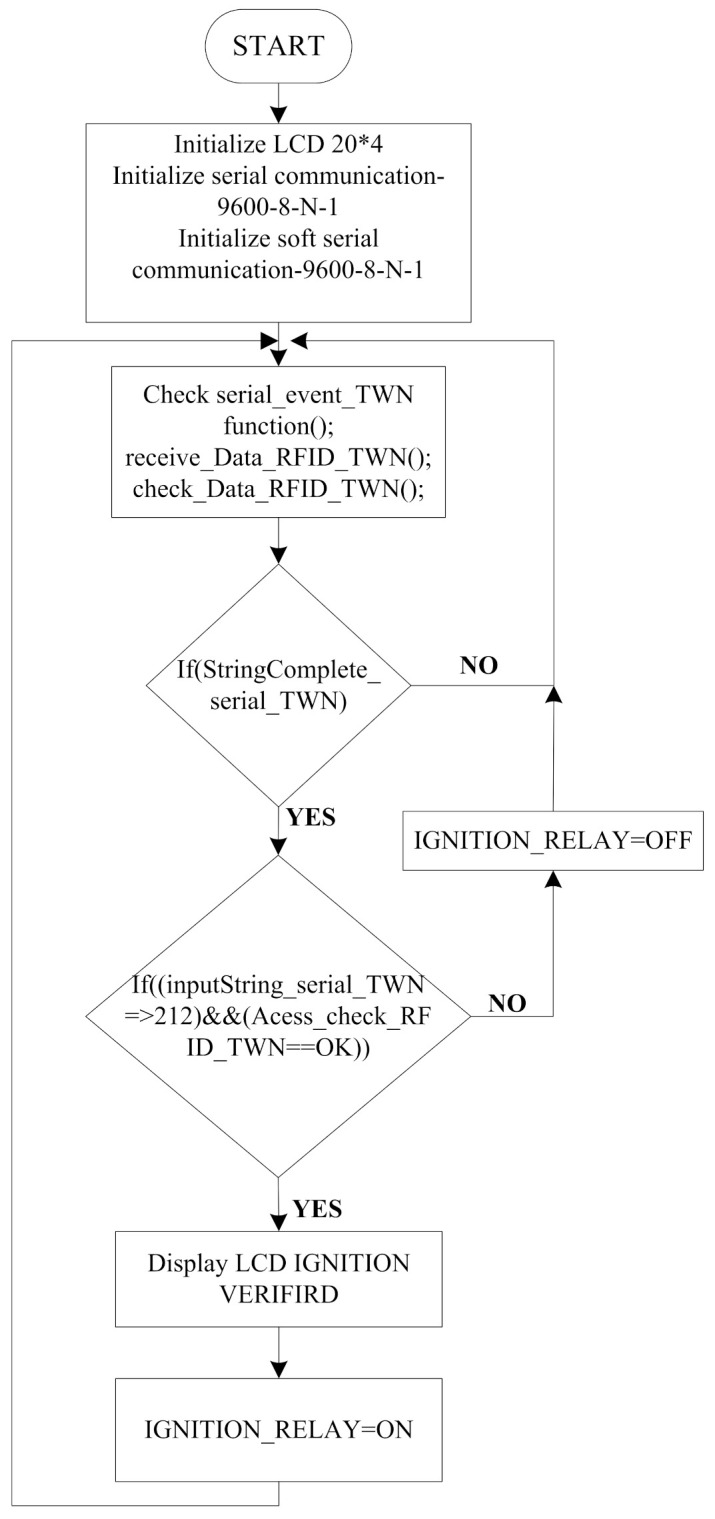
Flow chart for the two-wheeler node.

Figure 10 shows the flow chart for the two-wheeler node. The functions for LCD and serial communication are initialized. After the initialization, the system will receive RFID data when the card is swiped on the RFID reader and checks it by comparing the received data with the pre-defined code in the program. If it matches with the predefined code then a check is performed on the average value of flex sensors received from helmet node and RFID tag code, if both match with predefined values then the vehicle will be ignited, otherwise not.

**Figure 11:**
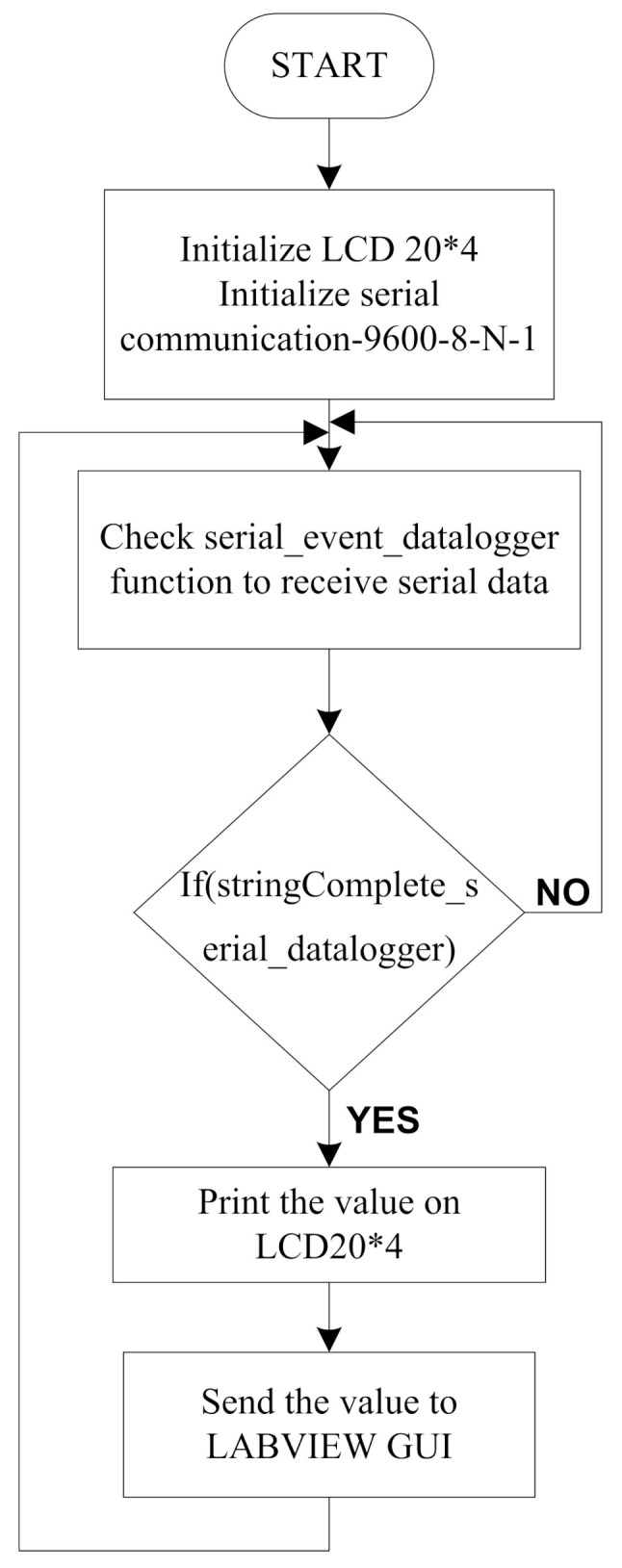
Flow chart for the LabVIEW GUI.

Figure 11 shows the flow chart for the LabVIEW GUI program. The functions for LCD and serial communication are initialized. Then, a check is performed for the serial events to receive data, if a complete string is received then it will be printed on the LCD and sent to the LabVIEW GUI, otherwise the system will again check for valid serial data.

## 6. Experimental Research

Experimental research for the system is done to ensure the selection of an appropriate experimental design. It involves the following steps, namely: define and state the problem, develop a hypothesis, design and conduct experiments to test the hypothesis, collect data, analyze the data, interpret the data, and conclude about the hypothesis and provide the relevant accession numbers.

Hypothesis testing: The following steps are involved in testing a hypothesis: formulate a hypothesis, set up a suitable significance level, choose a test criterion, compute the statistic from the samples, and make the decision

If a hypothesis is of the type µ = µ_H0_ then it is called a specific hypothesis but if it is of the type µ > µ_H0_, µ < µ_H0_ then it is called a composite or nonspecific hypothesis.

If results do not support the null hypothesis which means something else is true, then it is known as the alternative hypothesis.

*‘t’-test*

A *t*-test can be performed on the samples with less than thirty samples of the same type. For that null hypothesis to be defined first, and following the formulas for the *t*-test, the conclusion can be made.

(4) $$t=\frac{\bar{x}-\mu_{H0}}{\sigma_{s}/\sqrt{n}}$$

With a degree of freedom = (n − 1)

(5) $$\sigma_{s}=\sqrt{\frac{\sum {\left(x_{i}-\bar{x}\right)}^{2}}{n-1}}$$

### 6.1. Analysis for the Experimental Set Up

The concept of the null hypothesis with a *t*-test is applied for the analysis of statistical data collected from the samples and further assists to calculate the threshold value of flex sensors. The ten samples are collected from February 2020 and June 2020 at the University of Petroleum and Energy Studies, Dehradun with a temperature variation from 21 °C to 41 °C. The *t*-test is applied on the samples for calculating the threshold value of the sensor. *T*-test calculations are done with null hypothesis ‘212’ and further the calculation is done with a 1% level of significance for the *t*-test.

#### 6.1.1. Analysis for February 2020

In this section, analysis is carried out for February 2020 on the age group of 18–25 years with the temperature conditions of 21 °C to 27 °C. Moreover, the inbuilt analog-to-digital converter (ADC) of the Arduino controller with 10-bit resolution divides the output analog value of the flex sensor into 2^10^ (1024) levels. Every level is equal to 4.88 mV. The output voltage of sensors is converted into levels by the ADC of the Arduino controller and a further controller displays the sensor value on the display unit. Table 2 shows the sample data collected through the flex sensor in February 2020.

**Figure 12:**
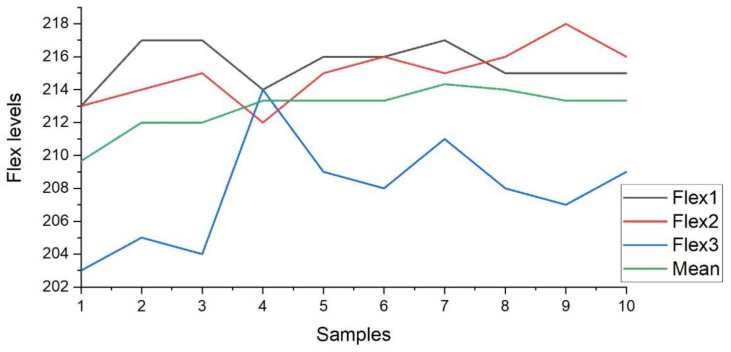
Value variations for samples in Feb 2020.

Furthermore, the Figure 12 presents the plot of the flex1, flex2, and flex3 sensors for the ten samples. The sample variation in the output level for February 2020 is visible in the plot. Figure 12 also describes the mean (green line in plot) of the three flex sensor values obtained from the ten samples.

The *t*-test is applied on the mean values of sensors mentioned in Table 2, and calculations are done with the help of standard formulas from Equation (4). The calculation of the *t*-test for the sensor’s value is mentioned in Table 3. The value calculated by the null hypothesis is less than the value from the table. Therefore, it is an acceptable hypothesis.

$\bar{x}$ is mean value of the samples = 212.86

The null hypothesis µH0 = 212

The value of ∑(xi − $\bar{x}$ )^2^ = 16.284

The value of σs = 1.345

Calculating 1% level significance, the value of ‘*t*’ = 2.02

The value from *t*-distribution table = 3.250.

#### 6.1.2. Analysis for June 2020

In this section, an analysis is carried out for June 2020 on the age group of 18–25 years with the temperature conditions of 34 °C to 41 °C. Furthermore, the Arduino controller’s inbuilt analog-to-digital converter (ADC) with 10-bit resolution differentiates the flex sensor’s output analog value into 2^10^ (1024) level. Every level has a voltage of 4.88 mV. Table 4 presents the sample data taken in June 2020 using a flex sensor.

**Figure 13:**
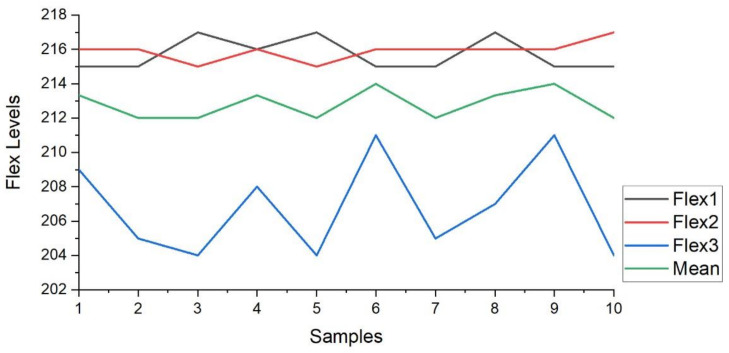
Value variations for samples in June 2020.

In addition, Figure 13 presents the plot of the flex1, flex2, and flex3 sensors for the ten samples. The sample variation in the output level for June 2020 is visible in the plot. Figure 13 also describes the mean (green line in plot) of the three flex sensor values obtained from the ten samples. The value calculated by the null hypothesis is less than the value from the tables, so it is an acceptable hypothesis. Therefore, the threshold value for flex sensor values on the helmet to ignite the vehicle, for the age group of 18–25 years, is calculated as ‘212’. The section concluded the analysis of the results for the experimental research with the help of LabVIEW GUI and samples collected. It is concluded that the vehicle will be ignited only if the average value from the three flex sensors exceeds level 212 (1.03 V) and the RFID code matches with the pre-stored RFID code to the program. If any one of these values is not satisfied, then the vehicle will not be ignited. To analyze the sensory data and ignition system, two LabVIEW GUI are designed. The major conclusion is in the form of a flex sensor-based system to ignite the vehicle only if the driver is wearing the helmet.

The *t*-test is performed on the mean values of the sensors listed in Table 4, and calculations are made using the standard formula from Equation (4). Table 5 shows the results of the *t*-test for the sensor values.

$\bar{x}$ is the mean value of the samples = 212.79

The null hypothesis µH0 = 212

The value of ∑(xi − $\bar{x}$ )^2^ = 8.0315.

The value of σ_s_s__ = 0.944.

Calculating 1% level significance, the value of *t_s_* = 2.674.

The value from *t*-distribution table = 3.250.

## 7. Results

In this section, we present the implementation of the proposed system in a real-time environment. Here the LABVIEW is utilized for analyzing and setting the threshold value in the system. Moreover, the three-flex sensor value is recorded in the cloud server is also presented in this section. Finally, the current consumption of the individual components is also included.

### 7.1. Prototype of the Designed System

**Figure 14:**
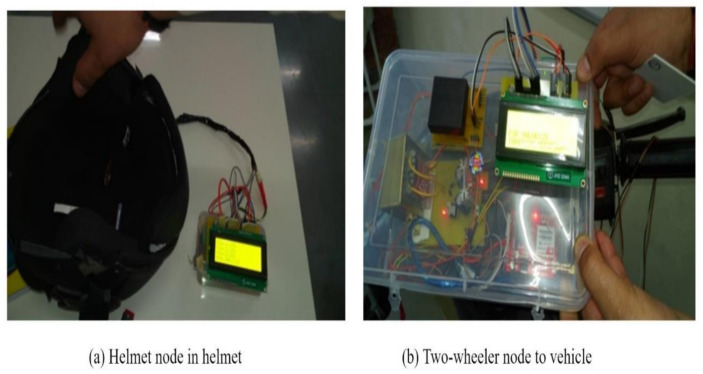
**The** helmet node and two-wheeler node.

**Figure 15:**
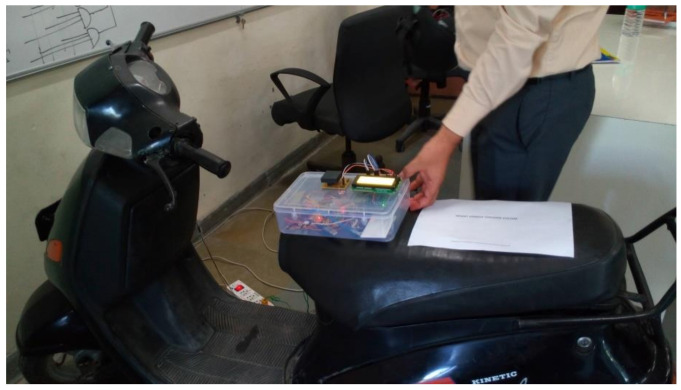
Hardware embedded to two-wheeler.

The proposed system is designed and developed for implementation in the real-time scenario to evaluate the performance. The helmet node based on 2.4 GHz RF communication embedded in the helmet is shown in Figure 14a. The components are assembled and, with the help of LabVIEW GUI, the threshold value is analyzed and set in the system. The developed helmet node and two-wheeler node are integrated into the two-wheeler as shown in Figure 15. The helmet node will be worn by the individual and the two-wheeler node is integrated into the ignition of the two-wheeler.

**Figure 16:**
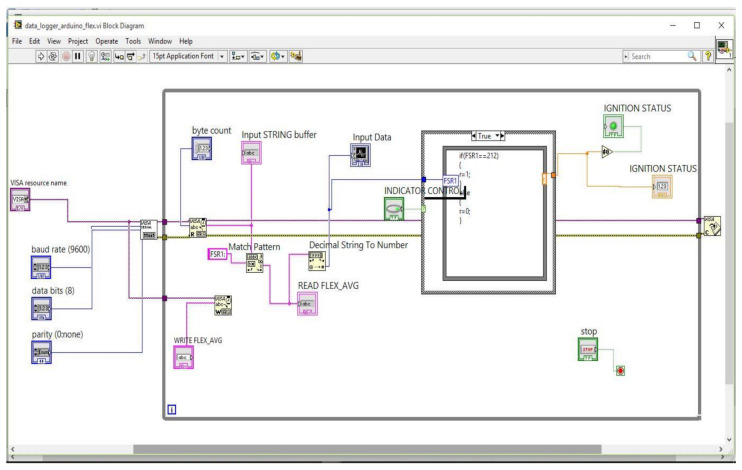
Block diagram for Lab VIEW for the system analysis.

**Figure 17:**
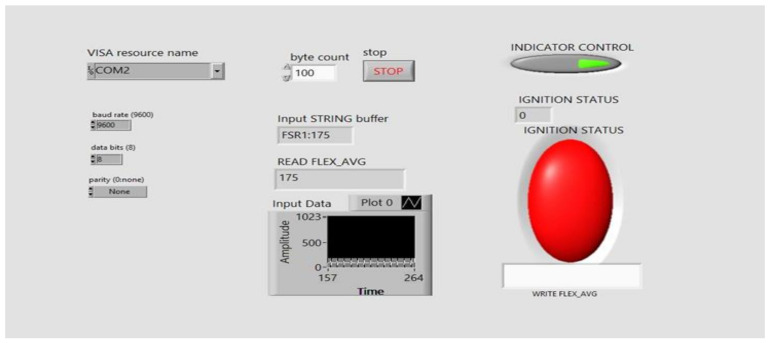
Front panel for Lab VIEW for system analysis showing the vehicle is not ignited.

Figure 16 shows the block diagram for the LabVIEW GUI designed to analyze the working of the system. Figure 17 presents the front panel of the LabVIEW GUI, where the two types of input indicator control loops are written in programming in such a way that if both the signals are matched with pre-defined values then the indictor shows green, otherwise red. Here, the green indicator signifies the ignition of the vehicle, and the red signifies that the vehicle is not ignited.

### 7.2. Sensory Data Analysis on Cloud Server

The global server is designed to analyze the sensor data. The importance of the “Internet of things” would be in terms of analyzing the sensory data from anywhere in the world. By creating a cloud server, sensory data is transmitted on the cloud through Node MCU. This can help generate an alert signal if the helmet pressure exceeds a threshold level, which indicates the driver has met with an accident. By checking the coordinates of the location on a cloud server, information can be sent to the nearest hospital or police station for quick help. In this study, through data analysis of each sensor, the node is used for calculating the threshold value of sensors to ignite the vehicle with experimental data, and sensory data is transmitted on the cloud through the “IoT”. The future scope of the work can be to calculate the threshold value of impact which can determine that an accident has occurred.

**Figure 18:**
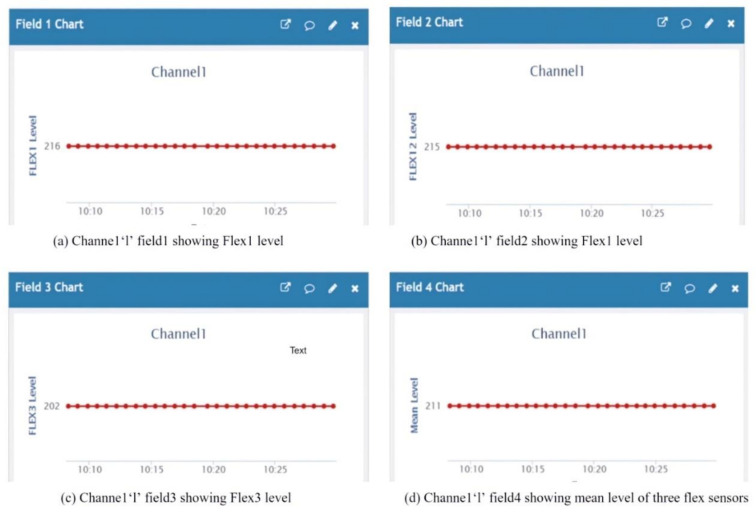
Channel ‘1’ showing the value of all three flex sensors with the mean level.

**Figure 19:**
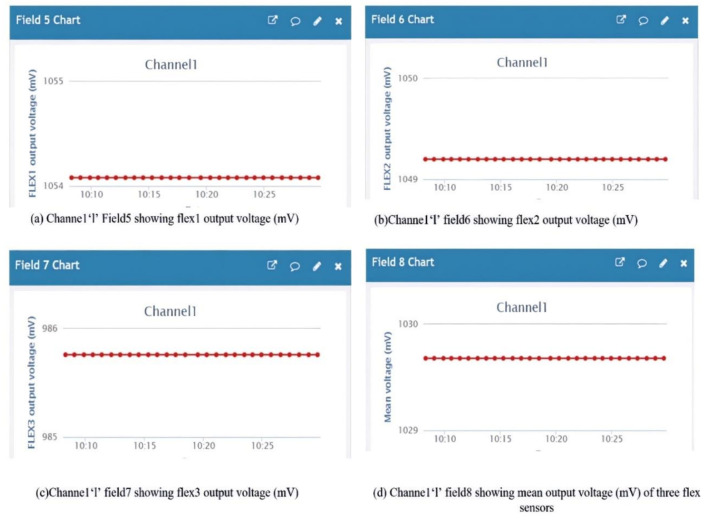
Channel ‘1’ showing the voltage output (mV) of all three flex sensors with mean level.

Data analysis is done for each flex output and the mean values in terms of level and voltage w.r.t time. Figure 18a shows the flex 1 level as ‘216’ at field 1 of the channel ‘1’ chart and Figure 18b the flex 2 level as ‘215’ at field 2 of the channel ‘1’ chart. Figure 18c shows the flex 2 level as ‘202’ at field 2 of the channel ‘1’ chart, and Figure 18d shows the flex 2 level as ‘211 ’at field the 2 of channel ‘1’ chart. Figure 19a shows the voltage output (mV) of flex 1 as ‘1054’ at field 5 of the channel ‘1’ chart and Figure 19b shows the voltage output (mV) of flex 2 as ‘1049’ at field 6 of the channel ‘1’ chart. Figure 19c shows the voltage output (mV) of flex 3 as ‘986’ at field 7 of the channel ‘1’ chart and Figure 19d shows the mean voltage output (mV) of all three flex sensors as ‘1035’ at field 8 of the channel ‘1’ chart.

### 7.3. Current Consumption Analysis

The battery current system is a rechargeable Lithium-Ion battery with a capacity of 12 V/1 A; hence it can be used (day/night) continuously in the system for around 10.05 h. The total power consumption by the helmet node is (99.5 mA × 5 V = 497.5 mW) the two components that dominate the power consumption helmet node are RF modem and Arduino, as shown in Table 6.

The total power consumption by the two-wheeler node is 188 mA × 5 V = 940 mW. The three components that dominate power consumption in the two-wheeler node are the RF modem, RFID reader, and Arduino, as shown in Table 7. The battery available in the vehicle is used to provide current to the developed system.

The total power consumption by the helmet node is 98 mA × 5 V = 490 mW. The two components that dominate the power consumption of the helmet node are the RF modem and Arduino, as shown in Table 8. The power can be taken from the main power supply at homes or industries.

## 8. Conclusion and Future Scope

Wearing a helmet protects the driver of the two-wheeler from any head injuries in case of an accident. Around 37% of deaths in accidents are caused by the driver not wearing a helmet. This study aims to improve the safety of the driver by integrating advanced technologies. Here, a customized helmet node and two-wheeler node is developed and deployed for controlling the ignition and authentication of the vehicle through RFID technology and 2.4 GHz RF communication. The threshold value for the ignition of the vehicle is determined with the *t*-test hypothesis on the experimental data. The threshold value is calculated as ‘212’ or 1.03 V. The total power consumption by the two-wheeler node and helmet node is calculated as 940 mW and 490 mW, respectively. In future, the study on the interference of RF signals of the same frequency band may be carried out. The study on the impact of an accident and action by alert system is another future scope of the present work. The real-time data can be used for applying predictions to enhance the system and make it more intelligent.

## Figures and Tables

**Table 1:** Comparison of proposed study with existing studies.

Ref.	Function	Real-Time Hardware	Ignition Mechanism	User Authentication	Communication	Threshold Value for Detection	Proof of Concept
[7]	Detection of and tracking of two-wheeler during an accident	Real-time hardware is implemented without customization	Power off the ignition through the accelerometer sensor	The limit switch is useful for detecting the user	GSM for communicating messages and GPS for updating the location	The threshold value is not carried out during the development of the system	Tested in a real-time environment.
[19]	Convolutional object detector for detecting non-helmeted motorcyclists at a traffic light	Software and camera-based is implemented	The ignition mechanism is not covered	Authentication of two-wheeler is missing	A request-response protocol act as a medium for routing the videos to the server.	The proposed system is based on the video, so the threshold value is set based on trained images	Yes, implemented in real-time.
[34]	Real-time detection of blunt-force impact events on helmets for every individual	Fiber Bragg grating (FBG) sensor is integrated with the customized helmet	The focus of the study is on detecting the blunt force impact on the head. So ignition is not covered in the study	NA	Wireless FBG transceiver is for sending the transient signals	The magnitude and direction of the impact event are provided via transient signals.	Bowling ball Pendulum Impactor System (PIS) was constructed and employed for simulating concussive events
[36]	a drowsy driver alert is implemented with the Video Stream Processing (VSP).	Raspberry pi 3 modules are implemented as hardware	The proposed system is limited to the detection of driver drowsiness	User identification is processed with the assistance of an eye	Wi-Fi inbuilt in Raspberry Pi3	Vision-based information is considered	Implemented the system in real-time with hardware.
[37]	Vehicle tracking and accident alert of the car	Fingerprint and Node MCU hardware is integrated	The fingerprint sensor is used for igniting the vehicle	Fingerprint-based user authentication	Wi-Fi is available in Node MCU.	A threshold value is not required.	Integrated the system in the vehicle for enabling ignition and authentication
Proposed	Authentication of wearing a helmet for igniting the two-wheeler.	Hardware is realized for real-time implementation	Flex sensor and RFID	RFID tag	2.4 GHz RF communication to connect helmet node with two-wheeler node and Wi-Fi to connect to the server	the *t*-test is applied on RAW flex sensor value on different samples	The developed system is implemented on two-wheeler vehicles.

**Table 2:** The output of three flex sensors in analog and voltage with mean (Age group: 18–25 years, temperature 21 °C to 27 °C, February 2020).

Samples	Flex 1 (Analog)	Flex 1 (Voltage)	Flex 2 (Analog)	Flex 2 (Voltage)	Flex 3 (Analog)	Flex 3 (Voltage)	Mean (Analog)	Mean (Voltage)
1	213	1.04	213	1.04	203	0.99	209.66	1.024
2	217	1.06	214	1.05	205	1.001	212	1.03
3	217	1.06	215	1.05	204	0.99	212	1.03
4	214	1.04	212	1.03	214	1.04	213.33	1.042
5	216	1.05	215	1.05	209	1.021	213.33	1.042
6	216	1.05	216	1.055	208	1.01	213.33	1.042
7	217	1.06	215	1.05	211	1.03	214.33	1.047
8	215	1.05	216	1.055	208	1.01	214	1.04
9	215	1.05	218	1.06	207	1.01	213.33	1.042
10	215	1.05	216	1.055	209	1.021	213.33	1.042

**Table 3:** *t*-test calculation on the samples collected in February 2020.

S.No.	Samples	**(xi −** $\bar{x}$ **)**	**(xi −** $\bar{x}$ **)^2^**
1	209.66	−3.2	10.24
2	212	−0.86	0.7396
3	212	−0.86	0.7396
4	213.33	0.47	0.2209
5	213.33	0.47	0.2209
6	213.33	0.47	0.2209
7	214.33	1.47	2.1609
8	214	1.14	1.2996
9	213.33	0.47	0.2209
10	213.33	0.47	0.2209

**Table 4:** The output of three flex sensors in analog and voltage with mean (Age group: 18–25 years, temperature 34 °C to 41 °C, June 2020).

Samples	Flex 1 (Analog)	Flex 1 (Voltage)	Flex 2 (Analog)	Flex 2 (Voltage)	Flex 3 (Analog)	Flex 3 (Voltage)	Mean (Analog)	Mean (Voltage)
1	215	1.05	216	1.055	209	1.021	213.33	1.042
2	215	1.05	216	1.055	205	1.001	212	1.03
3	217	1.060	215	1.05	204	0.99	212	1.03
4	216	1.055	216	1.055	208	1.016	213.33	1.042
5	217	1.06	215	1.05	204	0.99	212	1.03
6	215	1.05	216	1.055	211	1.03	214	1.04
7	215	1.05	216	1.055	205	1.001	212	1.03
8	217	1.06	216	1.055	207	1.01	213.33	1.042
9	215	1.05	216	1.055	211	1.03	214	1.04
10	215	1.05	217	1.055	204	1.021	212	1.042

**Table 5:** *t*-test on the samples collected in June 2020.

S.No.	Samples	**(xi −** $\bar{x}$ **)**	**(xi −** $\bar{x}$ **)^2^**
1	213.33	0.198	0.039204
2	212	−1.132	1.281424
3	212	−1.132	1.281424
4	213.33	0.198	0.039204
5	212	−1.132	1.281424
6	214	0.868	0.753424
7	212	−1.132	1.281424
8	213.33	0.198	0.039204
9	214	0.868	0.753424
10	212	−1.132	1.281424

**Table 6:** Current consumption analysis of the helmet node.

Component	Quantity	Current (mA)
Arduino nano	1	40
Flex Sensor	3	1.5
RF Modem	1	58
Total		99.5

**Table 7:** Current consumption analysis of the two-wheeler node.

Component	Quantity	Current (mA)
Arduino Uno	1	40
RFID reader	1	90
RF Modem	1	58
Total		188

**Table 8:** Current Consumption Analysis of Server.

Component	Quantity	Current (mA)
Arduino nano	1	40
RF Modem	1	58
Total		98

## Data Availability

Not applicable.

## References

[1] Motorized Two-Wheelers in Indian Cities | WRI Ross Center for Sustainable Cities, (n.d.). https://wrirosscities.org/research/publication/motorized-two-wheelers-indian-cities.

[2] India: Vehicle Production Volume by Segment 2021 | Statista, (n.d.). https://www.statista.com/statistics/607818/vehicle-production-volume-by-segment-india/.

[3] Mohan D. (2009). Road Accidents in India. IATSS Res..

[4] WHO (2004). Road Safety—Helmet Fact Sheet.

[5] Mohan D., Jha A., Chauhan S.S. (2021). Future of road safety and SDG 3.6 goals in six Indian cities. IATSS Res..

[6] Savino G., Lot R., Massaro M., Rizzi M., Symeonidis I., Will S., Brown J. (2020). Active safety systems for powered two-wheelers: A systematic review. Traffic Inj. Prev..

[7] Varade A., Gajbhiye N., Panchbhai V. (2017). Smart Helmet using GSM and GPS. Int. Res. J. Eng. Technol..

[8] Paiva S., Ahad M., Tripathi G., Feroz N., Casalino G. (2021). Enabling Technologies for Urban Smart Mobility: Recent Trends, Opportunities and Challenges. Sensors.

[9] Sargun S.M., Rana D.S.B. (2015). Wireless Personal Area Networks architecture and protocols for multimedia applications. Int. J. Adv. Res. Comput. Commun. Eng..

[10] Joy S.P., Sunitha V.S., Devi V.R.S., Sneha A., Deepak S., Raju A.J. (2016). A novel security enabled speed monitoring system for two wheelers using wireless technology. Proceedings of the 2016 International Conference on Circuit, Power and Computing Technologies (ICCPCT).

[11] Mahajan N., Kaur J. (2015). A Review of 2.4 GHz Transmitters for IEEE 802.15.4 Low Rate WPANs. Proceedings of the 2015 Second International Conference on Advances in Computing and Communication Engineering.

[12] Choudhury S., Kuchhal P., Singh R. (2015). Anita ZigBee and Bluetooth Network based Sensory Data Acquisition System. Procedia Comput. Sci..

[13] Shahare B., Chawde S., Gudafwar R., Pal H., Bobade P., Bawankar S. (2021). IoT based Smart Motor Cycle Helmet. J. Electron. Inform..

[14] Qian Y., Wu D., Bao W., Lorenz P. (2019). The Internet of Things for Smart Cities: Technologies and Applications. IEEE Netw..

[15] Alian S., Baker R., Wood S. (2016). Rural casualty crashes on the Kings Highway: A new approach for road safety studies. Accid. Anal. Prev..

[16] Gehlot A., Kuchhal P., Singh A., Singh R. (2017). Development and Analysis of FSR and RFID Based Authentication System. Advances in Intelligent Systems and Computing.

[17] Swathi S.J., Raj S., Devaraj D. (2019). Microcontroller and Sensor Based Smart Biking System for Driver’s Safety. Proceedings of the 2019 IEEE International Conference on Intelligent Techniques in Control, Optimization and Signal Processing (INCOS).

[18] Sreeja B.P., Manoj Kumar S., Sriram S. (2021). Professor, Smart Helmet for Alcohol Detection to Prevent from Accidents. http://annalsofrscb.ro.

[19] Samuel S., Reghunadh S., Ashwin M.K., Sabu S., Nair S.S., Varghese R.R. (2020). An Intelligent Traffic Monitoring System for Non-Helmet Wearing Motorcyclists Detection. Proceedings of the 2020 International Conference on Data Analytics for Business and Industry: Way Towards a Sustainable Economy (ICDABI).

[20] Hollinger A., Wanderley M.M. (2006). Evaluation of commercial force-sensing resistors. Proceedings of the Proceedings of the International Conference on New Interfaces for Musical Expression.

[21] Buckley L., Bingham C.R., Flannagan C.A., Carter P.M., Almani F., Cicchino J.B. (2016). Observation of motorcycle helmet use rates in Michigan after partial repeal of the universal motorcycle helmet law. Accid. Anal. Prev..

[22] Rasli M.K.A.M., Madzhi N.K., Johari J. (2013). Smart helmet with sensors for accident prevention. Proceedings of the 2013 International Conference on Electrical, Electronics and System Engineering (ICEESE).

[23] Fernandes F.A.O., De Sousa R.A. (2013). Motorcycle helmets—A state of the art review. Accid. Anal. Prev..

[24] Ganti L., Bodhit A.N., Daneshvar Y., Patel P.S., Pulvino C., Hatchitt K., Hoelle R.M., Peters K.R., Kuchibhotla S., Lottenberg L. (2013). Impact of Helmet Use in Traumatic Brain Injuries Associated with Recreational Vehicles. Adv. Prev. Med..

[25] Rana N.K. (2009). Application of force sensing resistor (FSR) in design of pressure scanning system for plantar pressure measurement. Proceedings of the 2009 Second International Conference on Computer and Electrical Engineering.

[26] Singh R., Mishra S., Joshi P. (2011). Pressure monitoring in wireless sensor network using Zigbee transceiver module. Proceedings of the 2011 2nd International Conference on Computer and Communication Technology (ICCCT-2011).

[27] Sung W.-T., Hsu Y.-C. (2011). Designing an industrial real-time measurement and monitoring system based on embedded system and ZigBee. Expert Syst. Appl..

[28] Zhu H., Maalej N., Webster J., Tompkins W.J., Bach-Y-Rita P., Wertsch J. (1990). An umbilical data-acquisition system for measuring pressures between the foot and shoe. IEEE Trans. Biomed. Eng..

[29] Sarathkumar D., Kumar C.S., Nithya S., Thilagavathi E. (2016). Automatic Two Wheeler Driving Licence System by Using Labview. Int. J. Adv. Res. Electr. Electron. Instrum. Eng..

[30] Dhomne S., Bulkunde P., Lohakare S., Ukey A., Gajbiye S., Deolekar S., Shambarkar N. (2019). Smart Ignition System in Automobile Industries. IJIRT J..

[31] Shankar V., Mannering F. (1996). An exploratory multinomial logit analysis of single-vehicle motorcycle accident severity. J. Saf. Res..

[32] Zhao Y., Liu Y., Ni L.M. (2007). VIRE: Active RFID-based Localization Using Virtual Reference Elimination. Proceedings of the 2007 International Conference on Parallel Processing (ICPP 2007).

[33] Chandran S., Chandrasekar S., Elizabeth N.E. (2016). Konnect: An Internet of Things(IoT) based smart helmet for accident detection and notification. Proceedings of the 2016 IEEE Annual India Conference (INDICON).

[34] Zhuang Y., Yang Q., Han T., O’Malley R., Kumar A., Gerald R.E., Huang J. (2021). Fiber optic sensor embedded smart helmet for real-time impact sensing and analysis through machine learning. J. Neurosci. Methods.

[35] Gehlot A., Singh R., Samkaria R., Choudhury S. (2015). WPAN and PSO based Water Quality Monitoring with LabVIEW as data logger. Int. J. Eng. Technol. IJET.

[36] Biswal A.K., Singh D., Pattanayak B.K., Samanta D., Yang M.H. (2021). IoT-Based Smart Alert System for Drowsy Driver Detection. Wirel. Commun. Mob. Comput..

[37] Balaji S., Gumber A., Santhakumar R., Kumar M.R., Tiwari A., Debnath H. (2020). An IOT-Based Vehicle Tracking System with User Validation and Accident Alert Protocol. Soft Computing for Problem Solving.

[38] Huang J., Liu B., Jin H., Liu Z. (2018). WiAnti: An Anti-Interference Activity Recognition System Based on WiFi CSI. Proceedings of the 2018 IEEE International Conference on Internet of Things (iThings) and IEEE Green Computing and Communications (GreenCom) and IEEE Cyber, Physical and Social Computing (CPSCom) and IEEE Smart Data (SmartData).

[39] Gu Y., Ren F., Li J. (2016). PAWS: Passive Human Activity Recognition Based on WiFi Ambient Signals. IEEE Internet Things J..

